# Idiopathic pleuroparenchymal fibroelastosis: first case report in Lebanon

**DOI:** 10.1093/jscr/rjab355

**Published:** 2021-11-29

**Authors:** Samer Dbouk, Nagham Bazzi, Sally Mansour, Aly Masry, Bassam Mansour

## Abstract

Idiopathic pleuroparenchymal fibroelastosis (IPPFE) was initially described by Amitani *et al*. in Japan. It is characterized by visceral pleural fibrosis and adjacent lung parenchymalfibroelastosis with striking upper lobe predominance. Because of its rarity and the lack of clear diagnostic criteria, the prevalence of the disease is still unclear. We report the first case of IPPFE in Lebanon and the second one in the Arab World. A 37-year-old Iraqi man was admitted to the hospital with progressive dyspnea on exertion occurring since 2 years and associated with dry cough. Histo-pathologic results reported a prominent interstitial fibrosis mainly in upper parts, and no granulomatous tissue was detected. Therefore, the diagnosis of IPPFE was made. The IPPFE is a distinct entity that requires meticulous clinico-pathological correlation for an adequate diagnosis and a close follow-up as this entity can progress into more advanced stages.

## INTRODUCTION

Idiopathic pleuroparenchymal fibroelastosis (IPPFE) dates back to 1992 when it was described by Amitani *et al*. in Japan and was given its current term in 2004 by Frankel *et al*. [1, 2] As its name indicates, it is characterized by visceral pleural fibrosis and adjacent lung parenchymal fibroelastosis with a striking upper lobe predominance [1]. Nowadays, pleuroparenchymal fibroelastosis (PPFE) is considered as a single entity with high suspicious clinical and radiological nuances [3]. In the literature, scarce articles are reported about PPFE, but an increased awareness is witnessed among physicians about the disease.

Because of its rarity and the lack of clear diagnostic criteria, the prevalence of the disease is still confusing. Cheng *et al*. reported a bimodal age onset ranging from 13 to 87 years of age with a first peak in the third decade and a second one in the sixth decade and with a mean age of 53 years of age [1, 3]. The disease is not risk-free and can lead to progressive volume loss of the upper lobes, resulting in irreversible respiratory failure and death [4]. The commonest clinical features reported are: progressive breathlessness and cough; nonspecific chest discomfort and pleuritic pain, which is rarely persistent in the absence of pneumothorax [4]. We report the first case of IPPFE in Lebanon and the second one in the Arab World.

## CASE PRESENTATION

**Figure 1:**
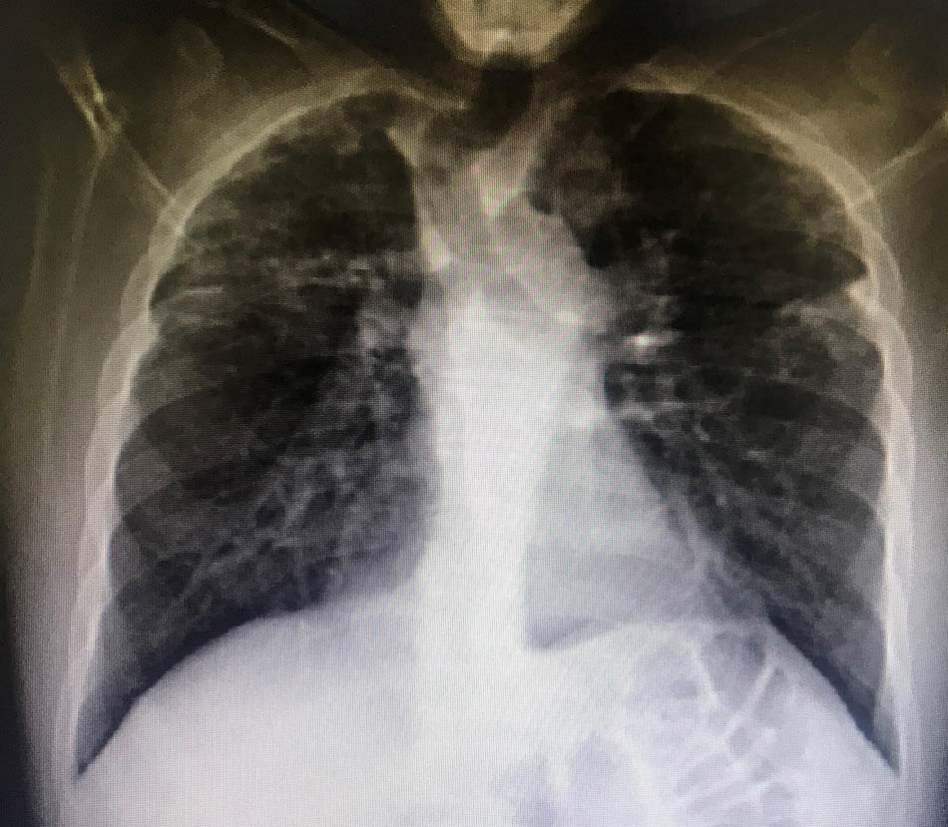
Left pneumothorax in a 37-year-old patient with IPPFE.

**Figure 2:**
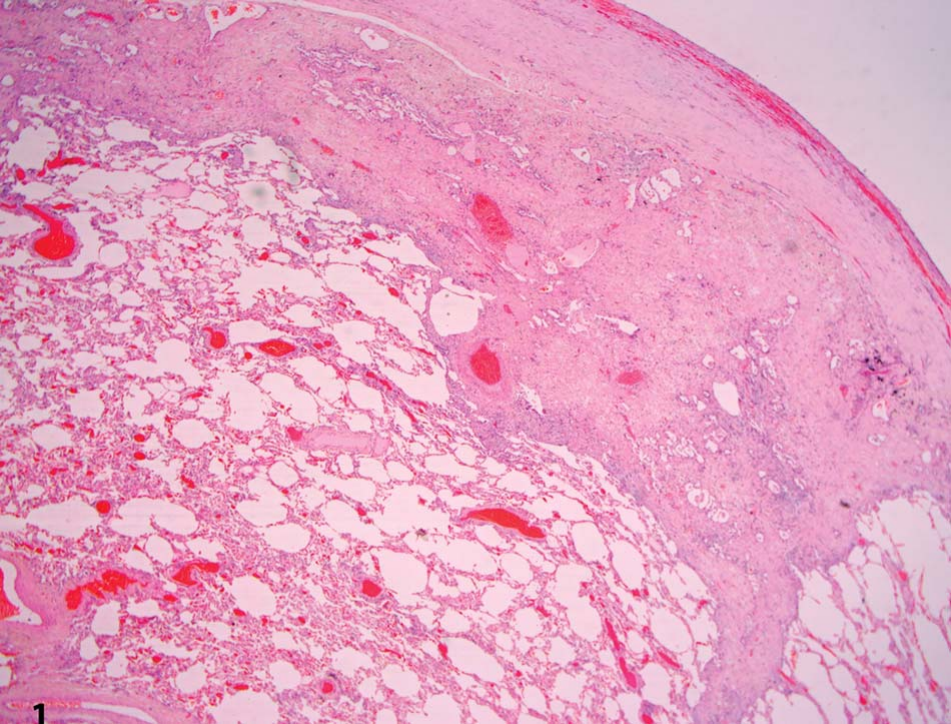
Low-power view showing dense pleural fibrosis associated with subjacent intra-alveolar fibrosis and accompanying septal elastosis, the latter being better seen on elastic Van Gieson stain; note the lack of fibrosing process away from the subpleural region (hematoxylin–eosin, original magnification ×20).

A 37-year-old Iraqi man was admitted to the hospital with progressive dyspnea on exertion occurring since 2 years and associated with dry cough. Patient was afebrile. The patient reported two episodes of severe dyspnea and low oxygen saturation occurring 1 month and 2 years ago. In both exacerbations, patient’s chest X-ray revealed left pneumothorax, and a tube thoracostomy was performed subsequently (Fig. 1). The patient served in the military service and was also a carpenter. Patient’s body mass index was 21 and his basal oxygen saturation was 97%. Physical exam showed flattened thorax and hypophonesis accentuated on the leftside during auscultation. At the admission, patient’s laboratory results were: pH = 7.43, PCO2 = 40, PO2 = 84, white blood cells count = 5350, neutrophils = 60, lymphocytes = 30, monocytes = 5.8, eosinophile = 2.1, hemoglobin = 14.1, hematocrit = 40, creatinine = 0.4, TSH = 0.51, free T4 = 11.53. A trans-thoracic echocardiography was made and did not show any significant finding. The purified protein derivative test results, the antinuclear antibody panel test and the rheumatoid factor detection test were negative. A video-assisted thoracoscopic surgery was performed to decorticate a restrictive layer of fibrous tissue overlying the lung, additionally to a wedge biopsy taken from the right apex. Histo-pathologic results reported a prominent interstitial fibrosis involving the subpleural and deep lung parenchyma with limited area of normal tissue intercepted between them (Fig. 2). No gronulomatous tissue was detected. The scarring tissue had a dense fibroelastotic appearance without active fibroblastic foci. Occasional dilated airspaces and ectatic lymphatic spaces were noted in the subpleural fibrotic parenchyma. Therefore, the diagnosis of IPPFE was made, and the other possible differential diagnoses are discussed below.

## DISCUSSION

The differential diagnosis of the disease is wide and includes: usual interstitial pneumonia (UIP) due to idiopathic pulmonary fibrosis (IPF), nonspecific interstitial pneumonitis (NSIP), hypersensitivity interstitial pneumonitis, pleuropulmonary fibroelastosis and advanced fibrosing sarcoidosis. UIP typical computed tomography (CT) pattern consists of honeycomb stage with basal-predominant distribution [5]. Additionally, IPF occurs in patients older than 60 years of age [5]. Therefore, UIP due to IPF was excluded. The NSIP consists mainly of parenchymal fibrosis with intact pleura and a lower predilection, which was not consistent with patient’s histopathology [6].

The definitive diagnosis of PPFE relies on the histological and radiological findings in surgical lung biopsy and includes pleural thickening with subpleural fibrosis in the upper lobes [3, 5]. Amitani *et al*. considered chest flatness as a constitutional symptom for IPPFE [2]. The exact etiology of PPFE is still unclear, but it is highly linked to lung, bone marrow and hematopoietic cell transplantation, chemotherapy drugs, repeated pulmonary inflammatory damage, IPF and occupational exposure [3]. Additionally, PPFE runs in families and can be transmitted genetically [3]. Being a carpenter, the patient was probably exposed to triggering dust, increasing the risk of pulmonary disease.

There is no effective treatment for IPPFE; however, therapy with steroids, pirfenidone, immunosuppressants and *N*-acetylcysteine has been reported with limited effects [7, 8]. Therefore, lung transplantation should be considered [8]. Compared to IPF, IPPFE has lower incidence of lung cancer but has similar incidence of acute exacerbations. Due to its rapid progression, survival rate of IPPFE patients was lower than IPF patients, and the commonest cause of death in IPPFE patients was chronic respiratory failure [9].

## CONCLUSION

The IPPFE is a distinct entity that requires a meticulous clinico-pathological correlation for an adequate diagnosis and a close follow-up as this entity can progress into more advanced stages. Further cohort studies assessing available treatment modalities and the development of anti-fibrotic treatment are now warranted.
