# Corrigendum to: Idiopathic pleuroparenchymal fibroelastosis: first case report in Lebanon

**DOI:** 10.1093/jscr/rjac027

**Published:** 2022-01-25

**Authors:** Samer Dbouk, Nagham Bazzi, Sally Mansour, Aly Masry, Bassam Mansour

In the originally published version of this manuscript, there were errors made within in the authorship list.

This should read: “Samer Dbouk^†^, Nagham Bazzi^†^, Sally Mansour, Aly Masry, Bassam Mansour”, (where ^†^ indicates co-first authorship) instead of: “Nagham Bazzi^†^, Sally Mansour^†^, Aly Masry, Ahmad Shahrour, Bassam Mansour”.

These errors have now been corrected online.

